# Perspectives on Autism Spectrum Disorder Diagnosis, Symptoms, Treatment and Gender Roles: A Qualitative Study of Similarities and Differences between Sexes

**DOI:** 10.3390/ijerph20247183

**Published:** 2023-12-15

**Authors:** Sigrid Piening, Ingrid D. C. van Balkom, Anne Fleur Stapert, Daria Henning, Kirstin Greaves-Lord, Lineke R. C. D. Davids, Stynke Castelein, Yvonne Groen

**Affiliations:** 1 Autism Team Northern-Netherlands, Jonx, Department of (Youth) Mental Health and Autism of Lentis Psychiatric Institute, 9700 AB Groningen, The Netherlandsaf.stapert@lentis.nl (A.F.S.); k.greaves-lord@lentis.nl (K.G.-L.); 2 Outpatient Clinic for the Elderly, Lentis Psychiatric Institute, 9725 AG Groningen, The Netherlands; 3 Department of Clinical Psychology and Experimental Psychopathology, University of Groningen, 9712 TS Groningen, The Netherlands; 4 Medical Psychology, Martini Hospital, 9700 RM Groningen, The Netherlands; l.davids@mzh.nl; 5 Lentis Research, Lentis Psychiatric Institute, 9725 AG Groningen, The Netherlands; 6 Department of Clinical and Developmental Neuropsychology, University of Groningen, 9712 TS Groningen, The Netherlands

**Keywords:** autism spectrum disorder, gender role, qualitative research, life change events

## Abstract

This study aims to compare the experiences of women and men of different age groups with regard to their first autism spectrum disorder (ASD) diagnosis, symptoms, treatment, and gender roles to inform our understanding in clinical practice of differences as well as similarities. Semi-structured interviews were conducted amongst 22 women (*n* = 12) and men (*n* = 10) in three adult age groups regarding their diagnostic process, symptoms, treatment, and gender roles. Participants also filled out questionnaires on gender traits, social support, coping, and quality of life. Framework analysis guidelines were followed to identify subthemes within the three pre-defined key themes of the semi-structured interviews, and quantitative analyses were performed on the questionnaire results. Women often had caregiver roles and were more focused on social and family-oriented life aspects than men. Family and societal expectations may have been different for women from an early age onward and were considered burdensome by some, but not all. Views on ASD diagnosis, symptoms, and treatment were largely individually determined. The questionnaire results mostly showed no significant sex differences. Perceived gender roles differed between participants. In diagnosis and treatment, awareness of general gender differences and gender roles is important, but inter-individual differences and similar experiences in men should not be overlooked.

## 1. Introduction

The preponderance of men with autism spectrum disorder (ASD) compared to women [1,2] has been questioned in recent years, as evidence indicates that ASD may affect more women than previously assumed [3]. Since ASD screening instruments are largely based on male presentations, women may be overlooked [4,5] as they show less unusual and/or restricted interests [6].

Poor understanding of female ASD presentations and subsequent diagnostic delay or misdiagnosis can cause unmet clinical needs and comorbidity, affecting societal participation, costs, and quality of life [7,8]. Late-diagnosed women have expressed sadness when considering how different their lives could have been with a timely ASD diagnosis [9].

Research suggests expectations connected to gender roles may strongly emphasise social behaviours in girls [10,11,12,13] and contribute to camouflaging behaviours and better communicative abilities in women compared to men with ASD [11,14]. Camouflaging entails strategies and behaviours to appear less neurodivergent in social situations [15], while not changing or alleviating underlying ASD symptoms. Hull and colleagues subdivide camouflaging behaviour into three types: compensation, masking, and assimilation strategies, all intended to appear more neurotypical and fit in better [16]. Camouflaging requires conscious effort, can be very tiring, and may even be associated with suicidality [9,17,18].

Despite expanding research on ASD in women, unanswered questions remain [3]. For example, can symptom differences be (partly) attributed to sociocultural factors such as gender-related social roles, as [10] suggests in their study? Or might caregiver roles burden women with ASD and partly explain their fatigue? If there is a perceived burden, do age-related changes in social roles affect this [19,20]? And might coping styles, social networks, gender expression, and identity play a role? Moreover, similarities between women and men with ASD exist as well, e.g., self-reported empathy [21], level of impairment, sensory symptoms and cognitive functioning [22], and facial emotion perception [23]. Many men with ASD also use camouflaging techniques, begging the question of whether underlying individual characteristics might motivate camouflaging [14,24,25]. McQuaid and colleagues already suggested camouflaging might be more related to a late diagnosis than to biological sex [26]. General sex differences in health behaviour and symptom perception may exist as well [27,28]. Men with autism likely experience many of the same symptoms, but may tend to underreport and present only when symptoms have become overwhelming [29], in line with long-known general sex differences in health-related behaviours [30].

This study aims to qualitatively compare the experiences of both women and men of different age groups with regard to their process towards their first ASD diagnosis, symptoms, treatment, and especially their gender roles, to inform our understanding of differences as well as similarities. A better understanding of the gender difference in ASD symptoms, perceptions, and potential compensatory strategies will inform clinical practice, research, and the development of screening instruments that promote earlier identification and treatment of ASD in women.

## 2. Methods

### 2.1. Participants

Data were collected through semi-structured interviews and additional questionnaires. Inclusion was possible when aged ≥18 years and having an ASD classification according to DSM-5 criteria established by a qualified healthcare professional [31]. We aimed to interview both women (*n*~15) and men (also *n*~15) in early adulthood (18–34 years), middle adulthood (35–54 years), and late adulthood (55+), or as many participants as possible until reaching content saturation (where no new themes were being identified from the data). The exclusion criteria were insufficient proficiency in the Dutch language and a serious risk of psychological instability.

After interviewing twelve women and ten men, recruitment ceased as content saturation was reached with no new topics raised. The demographic characteristics of the participants are represented in Table 1.

### 2.2. Procedure

Recruitment took place in 2018–2019 by purposive sampling through healthcare professionals of potential participants at a specialised outpatient autism clinic in the Netherlands. Study information was provided to patients by their healthcare professionals. In the event that patients were interested in participating and gave permission and contact information, they were contacted by the researchers via email or telephone. As recruitment was carried out via healthcare professionals and kept anonymously, we are unable to report on numbers and reasons for refusal to participate. After study information and informed consent were discussed and arranged with participants, the interviews took place, either at the outpatient clinic or at the participants’ home. At the start of the interview, the participants and interviewers introduced themselves to each other, and participants were reminded about the aim of the study. Small breaks were held during the interview when requested by participants and/or deemed necessary by the interviewers, but no follow-up appointments were needed. No participants dropped out or revoked their informed consent during the process. Additional information, such as previous diagnoses, medication, and family setting, was obtained from the participants’ medical file with their permission. Information given during the interviews was not shared with the treating clinicians of the participants, and no treatment relationship existed between the participants and the interviewers at the time of the interview. All interviews were audio-recorded and conducted by two experienced healthcare professionals (DH and SP) with research experience and clinical treatment experience with people with ASD. One interviewer took the lead during the interviews, while the other interviewer made field notes, kept track of the remaining interview topics, and asked questions if clarification or more information was needed.

Fourteen interviews took place at the outpatient clinic and eight at participants’ homes. Due to the wide range of topics being discussed, interviews lasted two hours on average, with breaks being held during seven interviews. During three interviews, family members (e.g., parent, partner) were present upon request of the participants, sometimes adding to the answers given.

### 2.3. Instruments

#### 2.3.1. Semi-Structured Interview

The semi-structured interview guide was developed with clinicians, researchers, and experts through the experience of the study’s sounding board. Based on the literature, e.g., [3,10,14,17,19,32,33], three key themes were formulated: experiences with (1) the diagnostic process and treatment (both psychological and pharmacological), (2) ASD symptoms, and (3) gender roles. Each theme had a set of initial and probing questions, inviting participants to express and elaborate on their views. The semi-structured interview is available upon request.

#### 2.3.2. Questionnaires

Additionally, participants were invited to fill out the following questionnaires: the BEM sex role inventory (BSRI; measuring gender traits [34,35,36]); the ENRICHD social support inventory ESSI [37,38,39]; the Dutch Utrecht coping list (UCL) [40,41]; the World Health Organisation Quality of Life-Bref (WHOQoL-Bref) [42,43,44]; and two visual analogue scales (VAS) about gender identity and gender expression. Hard copy versions of the questionnaires were provided at the start of the interview and could be returned at a later date. When necessary, reminders were given after approximately two and three weeks. More information regarding the questionnaires, e.g., their validity and reliability, and the VAS scales can be found in the Supplementary Material (Instruments S1: gender differences in ASD).

No statistically significant differences were observed between female and male participants for any of the questionnaires, except for the BSRI Male scale (Table 2).

Mean scores on the BSRI androgynous scale did not appear to differ greatly between sexes. In accordance with their sex, women scored higher on the feminine scale and men scored higher on the masculine scale.

With the ESSI threshold for low perceived social support set at <18, the mean total scores on the ESSI cannot be considered low for both sexes. However, one male participant did perceive low social support, with a total score of 13.

Both sexes had very high mean scores on the UCL subscales avoidance and passive reacting, high scores on palliative reacting, and average scores on the subscales seeking social support and expression of emotion compared with their respective norm groups. On the subscale active Tackling, women had low scores, while men had average scores.

Mean scores on the WHOQoL-Bref indicated a mostly lower quality of life for the participants compared to the Dutch general population and psychiatric outpatient samples in previous research [44].

Cohen’s d results indicated non-existing or small effect sizes for the questionnaire (scale) results (ranging from d = 0.000 to d = 0.199), except for the BSRI female scale and the UCL reassuring thoughts scales (medium effects with d = 0.508 and d = 0.688, respectively) and the BSRI male scale as well as the UCL active tackling scale (large effects with d = 1.437 and d = 0.967, respectively).

**Figure 1:**
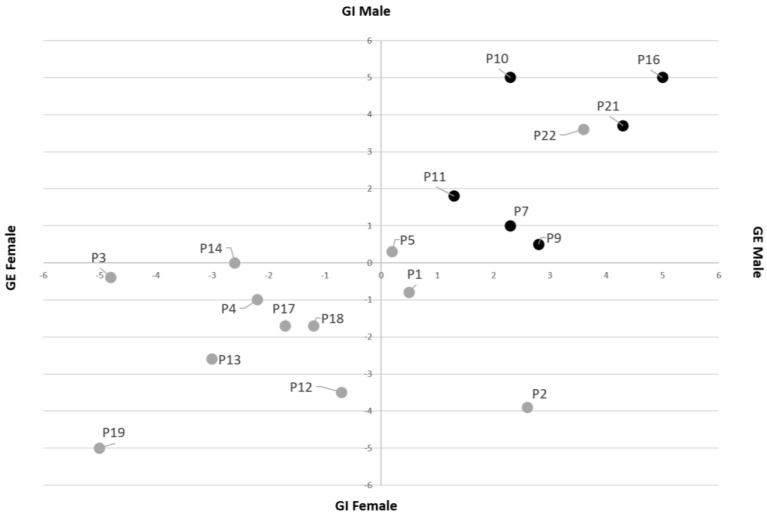
Gender expression and Gender Identity (*n* = 18). Note. GI = gender identity; GE = gender expression. Participants were asked to draw a cross on a horizontal line on how they perceive their own GI and GE. Feminine was indicated at the left side of the scale, neutral/androgynous in the middle and masculine at the right side of the scale. Both scales were combined to design Figure 1. Grey dots: women. Black dots: men. Missing data: four men (P6, P8, P15, P20). No one identified with a gender other than women or men.

On the VAS scales on gender identity and gender expression, men indicated feeling and expressing themselves as males (Figure 1). Women responded variedly, with four of 12 women expressing themselves as more masculine than feminine, and two of these four reporting a more masculine gender identity as well.

### 2.4. Data Analysis

The verbatim-transcribed interviews were imported into ATLAS.ti (Version 8; Scientific Software Development GmbH, Berlin, Germany) for qualitative data analyses. A second transcriber checked unclear parts of the audio recordings, and approximately ten percent of the transcribed text was randomly verified, after which a complete check was deemed unnecessary. During the coding of the transcripts, deductive and inductive approaches were combined using template analysis [45]. Coding was based on the interview topics, with the possibility to devise new codes when additional topics emerged. Coding was performed by one researcher and checked by a second researcher. Framework analysis guidelines were followed to identify themes within the three predefined key themes of the semi-structured interview [46]. Themes were discussed within the research team and adjusted when appropriate, followed by further interpretation of the meaning and significance of the data. Descriptive statistics, such as unpaired *t*-tests, Mann–Whitney U tests, and Cohen’s D, were used to calculate the results of the questionnaires comparing females and males using R Statistical Software (Version 4.1.2; R Core Team 2021) and SPSS (Version 26, IBM, Armonk, NY, USA).

### 2.5. Community Involvement Statement

Members of the study’s sounding board group, consisting of experts by experience, gave their advice throughout the study (e.g., the semi-structured interview topics and questions). A member of the project group is also an expert by experience (DJ, pseudonym).

## 3. Results

The experiences and views of participants are grouped into the following key themes: ASD diagnosis and treatment, life with ASD (ASD symptoms), and gender roles (including social support networks and expectations). Unless otherwise specified, results indicated with ‘participants’ represent results for participants of both sexes.

### 3.1. ASD Diagnosis and Treatment

**Something’s different, but what?**

Most participants felt different from an early age onward, often already in primary school. One participant even thought at that time:

*‘Am I from a different planet?’*

(Man, aged 18–34)

Some participants became aware of their differences at a later stage, e.g., after encountering difficulties at work or when recognising themselves in their children who were diagnosed with ASD. Some assumed that any differences lay with the outside world before realising their own behaviour or thought processes might be different.

**First diagnosis.**

Several participants reported that their complaints intensified so much over time that they needed help. After her children became more independent, one woman realised that a busy family life no longer explained her difficulties in getting things done. Participants sometimes sought help after significant life events and changes, such as the deaths of family members, work- or school-related problems, or international travel.

Some were triggered to wonder about possibly having autism themselves by books, articles, TV shows, acquaintances with ASD, or during professional training. However, often, either parents or healthcare professionals first thought of autism.

The time between first suspicion and first diagnosis was often lengthy for both sexes. One participant received mental health treatment for 15 years before his first ASD diagnosis, and all but two participants received their diagnosis after adolescence. Several older participants indicated that autism, especially in women, was relatively unknown when they were younger. Participants themselves sometimes had a stereotypical view of autism.

*‘I saw that movie “Rain Man” once. I never thought I could have that myself.’*

(Man, aged 35–54)

All but one participant reported previous and/or comorbid psychiatric diagnoses. One woman expressed frustration that her underlying ASD had previously been missed: *‘If they had diagnosed me correctly, a suitable treatment plan could have been chosen and maybe something could have been done about it. And now […], I’ve already had these [anxiety] complaints for ten years…’* (Women, aged 18–34)

**Hurray! I have ASD!**

After receiving the ASD diagnosis, most participants felt liberated, relieved, happy, or even euphoric: *‘Initially, I was kind of euphoric. Really hurray, happy. I thought: let’s put it in a big ad in the newspaper. […] Everyone should know.’* (Man, aged 55+)

Two men indicated no changes following the ASD diagnosis, although one of them thought the diagnosis could be beneficial for employers and job applicants (due to employer benefits for hiring employees with work limitations). In others, the diagnosis also instigated a process of grief and the realisation that life could have been different had they known earlier: *‘If I had known that I think and feel differently than most people, I could have handled that better. Many things wouldn’t have happened to me, or I would have reacted differently.’* (Women, aged 35–54)

Many participants reported subsequently learning more about ASD and what it meant for them individually: *‘I knew it. There’s a reason why I’m so tired. All those stimuli, I just can’t handle it.’* (Women, aged 35–54)

*‘It starts more like a foggy period in which everything doesn’t look that good or clear. And you don’t know exactly what the point of it is and how things go and what will come of it. But gradually it becomes clearer and every time you discover a new piece with which you can continue to expand on things that you could not put into words or express before. Well, and with that you get even more information and in the long run you reach a point at which you can say: yes, I can describe myself, I can express my problems and I can describe what I feel and what I don’t feel.’*

(Man, aged 35–54)

Learning about ASD included a process of (self) acceptance and managing a life with autism, such as deciding whom to inform. *‘But yes, well, how do you present that to the outside world, because everyone was already like: you’re weird.’* (Man, aged 35–54)

Most participants only informed immediate family members and close friends; others openly told new acquaintances. Participants considered reactions from others to be mostly neutral or even positive, with extra help being offered by relatives and teachers. However, negative experiences were also reported:

*‘[…] I got the response: Yes that is easy for you. You should not hide behind it. You should also adjust.’*

(Women, aged 55+)

*‘[…] and then it’s like: ‘yes, but everyone has something once in a while’. So yeah, I’ll keep it to myself.’*

(Women, aged 35–55)

**Positive and negative treatment experiences.**

Treatment ranged from outpatient consultations, e.g., cognitive behavioural therapy (CBT) and trauma therapy (EMDR), to psychiatric hospital admission. Experiences with treatment varied between individual participants, in particular with group therapy. Some considered psychoeducation and social skills training an opportunity to learn about others’ experiences, offering identification and normalisation, although the diversity of participants in group sessions often impeded this identification process. Pharmacotherapy was considered positive by participants and their social environment.

*‘[Without medication] I just can’t handle the world as well. I can’t process stimuli as well, I just react very rudimentary. Like a toddler actually, when I’m angry, I’ll stamp my feet. When I get sad, I’ll cry out loud.’*

(Women, aged 35–54)

*‘With the medication […] I suddenly remembered I was going to do certain things. Then I actually did what I had to do. And at the end of the day I was way more tolerable to be around. […] more manageable, more aware of what you are doing, a little more diplomatic in your interactions. […] Only, I don’t always actively notice it very much myself. It’s more that people don’t get angry with how you react or that you have a lot more energy left in the evening, more tolerable, you name it.’*

(Women, aged 18–34)

Frequent side effects of medication, sometimes leading to discontinuation, were noted for both sexes. When asked which treatment or insight had been most helpful, one participant mentioned learning to let go and accept things, while others found learning to express their difficulties to be most helpful.

*‘From time to time, if I remember something about my learning process, I put it in an email and I send it to one of my coaches. […] after some time I often receive a concisely formulated answer, which often increases my insight. That is a very pleasant process.’*

(Male, aged 55+)

Also indicated are getting to know oneself, lowering expectations somewhat, understanding the causes of sensory over- or under-stimulation, as well as finding meaning and usefulness in life. For some, treatment benefits were very clear; others found them harder to describe.

### 3.2. Life with ASD (ASD Symptoms)

**Advantages and challenges.**

Being able to focus on details was considered a talent by five participants of both sexes. Other self-reported strengths were being rational, honest, meticulous, loyal, dependable, very skilled at specific tasks, quiet, and calm. When asked about the disadvantages of ASD, participants reported that challenges with social communication and interactions, as well as sensory issues, executive functioning, and dealing with unexpected changes, were by far the most difficult aspects.

*‘I always compare it with a car navigation device: it calculates a route that you follow […] when you have to deviate the device says ‘recalculate’ and that takes a while. That’s kind of how it is with me.’*

(Women, aged 35–54)

*‘Body language is more difficult for me to understand.’*

(Man, aged 55+)

*‘It is indeed just purely on an emotional level and in communication with other people. There are certain things that I do not sense or very little, and other things I feel more strongly than others. […] Well, and often it is someone else’s emotional situation. If they don’t really tell me what is bothering them I often can’t really see what is going on and how serious it is.’*

(Male aged 35–54)

However, not everyone experienced similar difficulties.

*‘I have many friends of course. […] I was not that autistic person sitting on a bench alone without any friends. Outside I must say, I didn’t stay away from crackling tension, I liked it. […] I can also interact with someone at a party and then it turns into an animated conversation. I always say, I prefer to sit in bed with a blanket over my head. But still, I always manage the social stuff.’*

When asked about whether executive functioning is more difficult than social interaction in her case she said, *‘Yes, I think so. That’s why all those sticky notes are everywhere. That is absolutely true.’* (Women, aged 55+)

Anxiety, emotion regulation issues, rigidity, and rumination were reported by several participants.

*‘I always experience events three times. Beforehand I think about what might possibly happen, and then during… that’s what really happens, and then afterwards about what happened. That also takes a lot of energy.’*

(Women, aged 18–34)

**Social interaction, camouflaging, adapting to others and the cost of it all.**

Regarding social interactions, both sexes mostly reported difficulties in in-group interactions, anticipating every conversation in detail, and preferring to remain in the background. Other difficulties included small talk, phone calls, deducing others’ intentions, non-verbal communication, maintaining contact with friends and acquaintances, and dealing with uninteresting conversations. Only one woman reported no problems with social interaction.

Eye contact did not come naturally to both men and women but had usually been learned through observing others, relatives, TV, social skills training, or self-practice. Sometimes, participants applied different tactics, such as looking at people’s chins, to suggest attentiveness. Several participants considered direct eye contact too intense, causing them to become overwhelmed by the information or emotions of the other in question. Only one woman and one man reported no difficulties with eye contact.

Camouflaging behaviours were reported by both sexes. Three participants in the oldest age category spontaneously reported masking their behaviour to avoid standing out from the crowd while wanting to be perceived as strong, competent, and self-reliant. The remaining participants needed elaboration on the concepts of masking, imitating, and camouflaging. Subsequently, the majority recognised these aspects in their own behaviour, ranging from occasional imitation of others’ sentences to fully masking uncertainties during social interactions.

*‘Yes, I really was a chameleon… very good at adapting to group dynamics. I was also very good at exaggerating, which masked a bit of the insecurities I have.’*

(Man, aged 18–34)

*‘The trick is often just to act your way through life, but yes, that means you’re not really your true self.’*

(Man, aged 18–34)

Participants added that they wanted to be liked and wished to avoid potential conflict or disappointment with family members. Some camouflaging comes naturally by now; others reported doing it unconsciously or not adjusting behaviours at all during social interactions. While several participants noted the significant amount of energy social interactions might cost them, they indicated tiredness was mainly due to sensory stimuli but not necessarily to social contact itself.

**Daily activities.**

Participants’ daily activities consisted of general tasks related to education, work, family, volunteering, hobbies, or sports. The majority considered it likely that peers without ASD would be more sociable with others, less tired, and better at combining tasks/roles: *‘My fellow students are way more sociable and have active social lives besides their studies. I’m happy to be back home, it takes too much energy to do both.’* (Women, aged 18–34).

Two men said differences in daily activities compared to peers without ASD were mainly related to them being jobless but not necessarily to their ASD.

### 3.3. Gender Roles

**Social support networks.**

The social support networks of participants ranged from two family members to several siblings in combination with friends. Notably, while several women had (grand)children, none of the men had children. Men also lived alone more often, inherently reducing their caregiver roles. Important others were usually close friends, parents, siblings, and partners. Three women mentioned their (grand)children and three students mentioned their roommates and classmates. Relationships with parents and siblings ranged from close to troubled to non-existent. Preferences varied, with some being happy with their relatively small group and not minding time alone, and others wishing for larger networks. Four participants joined hobby clubs or volunteered at their church to prevent social isolation.

*‘I think that’s very important, because I know you need it. For otherwise you get lonely and socially isolated, if you don’t hang out with other people. But it does cost me a lot of energy. […] if it would cost me less energy […] I would consider it more important.’*

(Women, aged 18–34)

**Gender roles and expectations.**

Questions about which social role they considered most important in their lives resulted in distinguishable differences between sexes. Women most often mentioned a family-oriented role such as partner, mother, and student, while men answered variably, e.g., being themselves, a family member in general, or taking all roles at the same time. All women considered one of their family-related roles (e.g., mother, daughter, grandmother) to be their easiest role, while being an employee was the easiest for two men. *‘I think I’m strongest as an employee, [at work] is where I don’t ask that many questions and I am mostly goal oriented.’* (Man, aged 18–34).

The hardest role, according to some men, was being a partner or a son. Women answered more variably here, with one woman indicating that it was mainly combining every role in her life, which was the hardest for her. The expectations of others appeared to be burdensome to some women and men. Two women thought it was mainly their own high expectations of themselves that made life difficult. Five participants did not experience any problems with high expectations from their environment or themselves. As social roles changed over time, all participants had positive views on getting older. They felt more equal and more themselves without too much adaptation to others or to societal expectations.

**Partners, intimacy, and children.**

Quite a few participants were happy and positive about their partner and relationship. As many, however, reported difficulties, not considering themselves connected or good partners.

The men thought more positively about intimacy and sex than the women. While sex was enjoyed by three women, others considered sex less important, especially after childbearing. Sensory issues related to sex, such as hypersensitivities or environmental distractions, were sometimes mentioned as impeding factors.

Women with children reported that they enjoyed their pregnancies and emphasized the importance of their children. As none of the men had children, only women reported that parenting was often difficult or tiring. Two women reported that their parenting insecurities led them to (very) frequently ask advice from a professional or relative.

**Self-reported sex differences.**

All female participants reported that expectations during their upbringing emphasised social behaviour more for women than for men, which four men concurred with. Some men thought that women were generally better at small talk and expressing feelings than men.

*‘Women are, with all due respect, simply better at small talk. That is an assumption of mine, but if women are better at small talk, then they will also be more likely to mention such things, those little things they encounter. And men may be more likely to think, yes, it is serious, but yes, you know…there are indeed people like that… It will be more noticeable, because it is mentioned explicitly [by women].’*

(Male, aged 18–34)

Four participants considered men more autonomous and less eager to please than women: *‘Men didn’t care that much. ….. […] they could just be autistic, if you know what I mean.’* (Women, aged 55+).

Some participants thought differences were more individual than sex differences. Others indicated not knowing or seeing any differences between women and men with ASD.

## 4. Discussion

In this qualitative study of adults with ASD, we surprisingly found many similarities between men and women. The results mainly revealed individual but no sex differences regarding experiences with the ASD diagnosis, treatment, and symptoms, which changed with increasing age and life experience. However, perceived gender roles did differ between sexes, especially regarding gender expression and identity, the degree of family orientation, and socialisation.

### 4.1. ASD Diagnosis, Treatment, and Symptoms

Experiences with the process towards the first ASD diagnosis, symptoms, and treatment generally varied and were more individual than gender-related. The current findings largely correspond with the results of the growing body of studies on the experiences of women with autism [9,13,14,17,19,32,33,47,48,49]. For example, positive experiences with their ASD diagnosis, e.g., relief, understanding, and self-acceptance, concurred with previous reports, and similarly, comorbidity was the rule rather than the exception, impeding timely ASD diagnosis [9,17,19,32,33,48,49].

Similar to the women, some men also reported using camouflaging techniques. McQuaid and colleagues reported similar findings but additionally observed that women reported more camouflaging skills than men, which we cannot confirm due to our dissimilar study design [26]. Interestingly, McQuaids’ findings also indicated that late diagnosis might be related to slightly different symptom presentation with regard to camouflaging. With all but two participants being diagnosed after adolescence, this might be the case in the current study as well, and this aspect deserves more research.

While avoidant coping behaviours such as camouflaging can sometimes be useful in the short term, they are usually considered inappropriate coping styles. Avoidant coping styles appeared to be relatively high, and women especially had low rates of active coping. Avoidant coping is associated with decreased resilience [50], which in turn is associated with a lower quality of life for people with autism [51]. Consistent with the literature, we found the quality of life of the participants was below average as well [8,52]. Focusing on optimising coping styles during treatment might increase resilience and thus quality of life, although further research into these aspects seems indicated.

Interestingly, despite reporting social difficulties, all but one of the participants were satisfied with their social support network. Some participants deliberately kept their network relatively small, minimising the efforts needed to maintain it. As satisfaction with social networks has not been studied extensively in individuals with autism [53], the significance of these findings remains to be studied further.

All participants had positive views on becoming more experienced in life with age. Especially older participants felt more equal and more themselves without feeling the need to adapt to others or societal expectations as much anymore, suggesting increased resilience with age.

### 4.2. Gender Roles

Finally, yet importantly, the results appear to support the ideas of Goldman, Kreiser and White, and Eagly and Wood regarding the role of socialisation: women seemed more oriented towards caregiver, social, and family roles than men [10,11,54]. Although gender and gender roles have definitely changed in recent decades, moving from a mainly binary view of masculinity and femininity towards a more diverse construct in the present day [36], a traditional view of gender can still exist. Several participants of both sexes considered the upbringing of girls to be more focused on social behaviour, accompanied by more demanding family and societal expectations. Furthermore, the questionnaire suggested that the participants were highly similar regarding their gender traits, social support, most coping strategies, and quality of life. In spite of the possibly dated and culturally determined classification of gender-specific traits on the BSRI, women still scored highest on the feminine scale, as did men on the masculine scale. Statistically significant sex differences for the questionnaires were only observed for the BSRI Male scale, with women scoring significantly lower on this scale. In addition, a high effect size was observed for the BSRI Male scale, supporting the existence of differences on this scale despite the small sample size.

Women in our study reported more varied gender identities and expressions than men, corresponding with women in the general population and with the ASD population [33,49,55,56,57]. However, more research on identity development in general and related gender identity development in people with ASD is needed to clarify the underlying mechanisms of these differences. These results emphasise the importance of considering general differences in socialisation and gender roles in ASD symptom representation to be able to put differences into female-specific ASD symptoms in perspective. As Cheslack-Postava and Jordan-Young mentioned in their study, ‘*By omitting gendered socialization from the array of factors considered in producing M-F differences, researchers make the implicit assumption that it has no effect.*’ [12].

Jones and colleagues indicate that certain life events fit into a developmental process that neurotypical people may experience as well [58]. Seers and Hogg argue in their study that typical development should not be unjustly pathologized [49]. We wish to emphasise the current study certainly does not aim to minimise experiences of individuals, specifically women with ASD, whose quality of life may reportedly be lower than that of neurotypical individuals [8,52]. Several participants reported severe suffering at certain periods or even throughout their lives. We merely wish to indicate that gender-specific differences can also occur in people with ASD and should be taken into account in clinical practice and in research.

## 5. Strengths and Limitations

The semi-structured interview, designed collaboratively with experts based on experience and with themes based on literature was a major strength. Semi-structured interviewing allows for additional probing questions, which is advantageous for individuals with autism who may find open questions especially difficult to answer. As recommended by Lai and colleagues, in-depth interviewing of both sexes and sampling different age categories helped in identifying relevant issues in different life phases, which might not have emerged with quantitative study designs [3]. Smaller samples in qualitative studies may increase the risk of bias, which we have minimised by not setting IQ limits and by sampling different age categories. However, all but two of our participants were diagnosed with ASD at a later age. This might have biassed the results, but at the same time, it gives an impression of the challenges that late-diagnosed people with ASD encounter and should therefore not be dismissed. While not representative of all individuals with autism, especially those diagnosed early or without mental healthcare, the results point out the relevance of general gender differences independent of ASD, suggesting useful future research directions, e.g., comparing those with autism to those without autism [3].

The presence of parents/significant others during three interviews may have prompted socially desirable answers [59], although no obvious signs were detected and questions were primarily answered by participants. To gain and maintain a good working relationship with the participants, we respected the wishes of these participants to have their family members present during the interview. Additional information from others was transcribed as such and was considered important to support (self)reflection [13,60,61].

## 6. Recommendations

In clinical practice, it is important to be aware of general gender differences and gender-specific roles of women, but also not to rule out the possibility that men with ASD might have similar experiences. Some experiences can be considered typical and age-appropriate developmental tasks and should not be pathologized [49,58].

Stereotypical views on ASD were underlined by participants’ own initial views on ASD and subsequent lack of early recognition of ASD as the underlying explanation for their problems. Experienced experts on our Sounding Board indicated similar experiences with healthcare staff and others, with some being told their ability to make eye contact precluded an ASD diagnosis. Study participants reported being able to make eye contact although this was occasionally unpleasant. Understanding the considerable individual differences in ASD presentations and the impact stereotyped views have on early recognition is imperative.

Experiences with the gender roles of women and men with and without ASD can be further elucidated through a 2-factorial design, as was suggested by Lai and colleagues [3]. Future quantitative studies focusing on the female ASD phenotype should include aspect of gender roles. Lastly, investigating individual characteristics leading to late first diagnoses as well as the role of coping styles and age-related differences may improve access to appropriate services when needed and prevent misdiagnosis.

## 7. Conclusions

Gender roles differed between mainly late-diagnosed women and men participating in this study, but experiences with ASD diagnosis, symptoms, and treatment appeared to be largely individually determined and changed with increasing age and life experience. Quantitative studies on the role of gender differences and socialisation in individuals with ASD, both early and late diagnosed, are needed. In clinical practice, it is important to keep in mind the similarities between women and men with autism while at the same time increasing awareness of general gender differences and gender-specific roles, as well as variation in individual ASD presentation.

## Figures and Tables

**Table 1:** Demographic Characteristics.

*n* = 22	Women (*n* = 12)	Men (*n* = 10)
**Age**		
Mean age (years, SD)	40.3 (14.2)	40.5 (13.9)
18–34 (*n*)	4	4
35–54 (*n*)	5	3
≥55 (*n*)	3	3
**Age at first ASD diagnosis (*n*, %)**		
Adolescence (12–18 years)	1 (8)	1 (10)
Early adulthood (19–35 years)	5 (42)	5 (50)
Middle adulthood (36–55 years)	6 (50)	4 (40)
**Previous/comorbid diagnoses** (Y, %) ^A^	12 (100)	9 (90)
Mood disorders	5 (42)	5 (50)
Personality disorders	4 (33)	2 (20)
Anxiety disorders	4 (33)	1 (10)
Substance-related disorders	1 (8)	4 (40)
Other	8 (67)	3 (30)
**Psychotropic medication use** (Y, %)	9 (75)	7 (70)
Side effects	7 (58)	5 (50)
**Highest level of education attained (*n*, %)**		
Elementary education	0 (0)	1 (10)
Pre-vocational education (VMBO)	1 (8)	0 (0)
Higher general continued education (HAVO)	1 (8)	0 (0)
Preparatory scientific education (VWO)	3 (25)	0 (0)
Middle-level applied education (MBO)	5 (42)	6 (60)
Higher professional education (BSc)	0 (0)	2 (20)
Scientific education (MSc)	2 (17)	1 (10)
**Work status (*n*, %)** ^A^		
Student	1 (8)	1 (10)
Student + work	2 (17)	0 (0)
Employed (full- or part-time)	3 (25)	6 (60)
Unemployed	6 (50)	3 (30)
Receiving (illness or disability) benefits	7 (58)	6 (60)
Volunteer work	1 (8)	2 (20)
**Family member(s) with ASD (*n*, %)** ^A^		
Validated ASD classification (DSM 5)	4 (33)	4 (40)
Undiagnosed ASD symptoms observed by participant	11 (92)	4 (40)
**Living status (*n*, %)**		
Independent (with or without support)	2 (17)	7 (70)
With parent(s) (and siblings)	1 (8)	2 (20)
With partner and/or child(ren)	8 (67)	1 (10)
With housemate(s)/friend(s)	1 (8)	0 (0)

Note: SD = Standard deviation; ASD = Autism Spectrum Disorder; Y = Yes. ^A^ Several answers possible. Percentages may not total 100 due to rounding. No one identified with a gender other than women or men.

**Table 2:** Questionnaires.

					95% CI		
(*n* = 22)	Total	Female (*n* = 12)	Male (*n* = 10)	*p*-Value	Lower	Upper	Cohen’s d
**BSRI** Mean (*SD*)	*n* = 19 ^A^	*n* = 12	*n* = 7 ^A^				
BSRI-F	85.05 (10.15)	86.94 (9.33)	81.80 (11.41)	0.300 ^C^	−15.29	5.007	0.508
BSRI-M	78.25 (12.29)	72.85 (11.66)	87.51 (6.77)	0.008 ^C^	4.42	24.894	1.437
BSRI-A	83.87 (5.92)	84.26 (6.07)	83.21 (6.05)	0.719 ^C^	−7.141	5.03	0.173
**ESSI**	*n* = 19 ^A^	*n* = 12	*n* = 7 ^A^				
ESSI total score	24 (6.05)	24 (5.40)	25 (7.43)	0.682 ^C^	−4.789	7.439	0.161
**UCL** Mean (*SD*)	*n* = 16 ^A,B^	*n* = 9 ^B^	*n* = 7 ^A^				
Active tackling	16 (4.34)	14 (4.80)	18 (2.50)	0.515 ^C^	−3.882	7.454	0.967
Palliative reacting	21 (3.96)	21 (4.30)	21 (3.82)	0.432 ^C^	−7.495	3.352	0
Avoidance	20 (2.83)	20 (3.21)	20 (2.50)	0.797 ^D^	15.15	27.25	0
Seeking social support	12 (3.35)	12 (2.87)	11 (4.11)	0.243 ^C^	−8.358	2.262	0.298
Passive reacting	19 (3.38)	19 (3.81)	18 (2.97)	0.148 ^D^	13.26	29.34	0.283
Expression of emotion	6 (1.69)	6 (1.33)	6 (2.07)	0.864 ^D^	3.64	10.76	0
Reassuring thoughts	12 (3.02)	11 (2.71)	13 (3.24)	0.461 ^D^	8.51	15.29	0.688
**WHOQoL-Bref** Mean (*SD*)	*n* = 19 ^A^	*n* = 12	*n* = 7 ^A^				
Physical health	10.98 (2.19)	10.95 (2.46)	11.02 (1.83)	0.950 ^C^	−2.196	2.332	0.031
Psychological health	12.35 (2.08)	12.06 (2.37)	12.86 (1.48)	0.433 ^C^	−1.303	2.907	0.381
Social relationships	12.56 (2.89)	12.78 (3.09)	12.19 (2.71)	0.508 ^D^	9.71	15.49	0.199
Environment	14.63 (1.90)	14.54 (2.12)	14.79 (1.60)	0.796 ^C^	−1.713	2.201	0.128

Note: BSRI = BEM Sex-Role Inventory (BSRI-M = Masculine index; BSRI-F = Feminine index; BSRI-A = Androgyn index); ESSI = ENRICHED Social Support Inventory; UCL = Utrecht Coping List; WHOQoL-Bref = World Health Organization Quality of Life-abbreviated. ^A^ Missing: *n* = 3 (loss to follow-up). ^B^ Additional missing: *n* = 3 (accidental use of non-comparable version of UCL). ^C^ Unpaired *t*-test was used to compare group means of females and males. ^D^ Mann–Whitney U test was used to compare group means of females and males, as data for this variable were not normally distributed. None of the participants identified with a gender other than women or men.

## Data Availability

The data presented in this study are available on request from the corresponding author. The data are not publicly available due to privacy reasons.

## Supplementary Materials

The following supporting information can be downloaded at: https://www.mdpi.com/article/10.3390/ijerph20247183/s1, Instruments S1: Gender role experiences in ASD.
