# Supplementary material for: Perspectives on Autism Spectrum Disorder Diagnosis, Symptoms, Treatment and Gender Roles: A Qualitative Study of Similarities and Differences between Sexes
Source: Int J Environ Res Public Health. 2023 Dec 15;20(24):7183. doi: 10.3390/ijerph20247183 (PMC10742825; doi:10.3390/ijerph20247183)
Supplement: Supplementary file 1 [file ijerph-20-07183-s001.zip › ijerph-2721172-supplementary.pdf]

Supplementary Material S1: Gender role experiences in ASD

Quantitative questionnaires

Participants were invited to fill out the following questionnaires: the BEM sex role inventory (BSRI; measuring gender traits); ENRICHD social support inventory ESSI; the Dutch Utrecht coping list (UCL); the World Health Organisation Quality of Life-Bref (WHOQoL-Bref) and two visual analogue scales (VAS) about gender identity and gender expression.

BEM Sex Role Inventory (BSRI)

The BEM sex role inventory (BSRI) is an instrument that measures hypothesized gender traits [34]. The BSRI consist of three subscales (femininity, masculinity and androgynous) and 60 items with a seven point Likert scale ranging from 1 (never or almost never true) to 7 (always or almost always true). Respondents can score relatively high on the 20 traits that are considered to be feminine or on the 20 items that are considered to be masculine. The BSRI also contains the androgynous subscale with 20 more traits that are not considered to be specifically feminine or masculine.

The BSRI is a relatively old instrument (1974) and its usefulness is debated. It is to be expected that views on which traits are considered to be typical masculine or feminine change over time and can be culturally determined [35]. Moreover, it is a limited view on gender, where at the present day gender is regarded as a more diverse construct [36]. Nevertheless, the BSRI is still one of the most widely used instruments to assess gender roles and can still be considered a valid tool [35].

ENRICHD Social Support Inventory ESSI

The ENRICHD social support inventory (ESSI) is a seven item instrument to assess perceived social support [37]. The first six items are answered with a 5 point Likert scale ranging from 1 (none of the time) to 5 (all of the time). The last item is dichotomous and can be answered with either ‘yes’ (counted as 4 points) or ‘no’ (two points). The total score ranges from 8-34, with higher scores indicating greater perceived social support. The English version of the ESSI assumes low social support when scored < 18. [37]. The ESSI has been shown to have good validity and reliability [38,39]. The English version of the ESSI was translated into Dutch and backtranslated to ensure the meaning and intent of the original questions were guaranteed.

Utrecht Coping List (UCL)

The Dutch Utrecht coping list (UCL) is a widely used, validated questionnaire assessing coping strategies based on the Coping Scale by Westbrook and colleagues [40,41]. UCL domains are: active, palliative, avoiding, social support seeking, passive, expressing emotions, reassuring thoughts [40]. The UCL consists of 47 items with a four point Likert scale ranging from 1 ‘seldom or never’ to 4 ‘very often’. Higher scores on specific domains indicate stronger use of that coping style. Subdomain scores can be classified as either very low, low, average, high or very high, depending on the specific normgroup respondents are compared with.

World Health Organization Quality of Life – Bref (WHOQoL-Bref)

Quality of life (QoL) was assessed with the Dutch self-report version of the World Health Organisation Quality of Life – Bref (WHOQoL-Bref). The WHOQoL-Bref is a 26 item questionnaire using a five point Likert scale ranging from 1 ('disagree' or 'not at all') to 5 ('completely agree' or 'extremely') [42]. Higher scores indicate a higher QoL. Domains are: physical health, psychological, social relationships and environment, complemented with two questions on overall QoL and general health. Although the WHOQoL-Bref has no official normscores available, the instrument was extensively tested. It is considered 'a sound, cross-culturally valid assessment of QoL' and has shown good content validity, discriminant validity, internal consistency and test-retest reliability [42,43]. For this study, scores are compared with a sample of the general Dutch population and a Dutch psychiatric outpatient sample from the study by Masthoff and colleagues [44].

Gender Identity and Gender Expression Visual analogue scales

Two visual analogue scales (VAS) were designed for this study to assess gender identity (GI) and gender expression (GE) (available upon request). Gender identity was defined as one's own perceived gender. Gender expression was defined as the way one outwardly shows their gender identity by physical expression, clothing, hairstyle etc. Participants were asked to draw a cross on a horizontal line on how they perceive their GI and GE. Feminine was indicated at the left side of the scale, neutral/androgynous in the middle and masculine at the right side of the scale. Length in centimeters was used to calculate percentages of given answers.

References

34. Bem, S.L. The measurement of psychological androgyny. *J. Consult. Clin. Psychol.* **1974**, *42*, 155–162. https://doi.org/10.1037/h0036215.
35. Holt, C.L.; Ellis, J.B. Assessing the Current Validity of the Bem Sex-Role Inventory. *Sex. Roles* **1998**, *39*, 929–941. https://doi.org/10.1023/a:1018836923919.
36. Rubin, J.D.; Atwood, S.; Olson, K.R. Studying Gender Diversity. *Trends Cogn. Sci.* **2020**, *24*, 163–165. https://doi.org/10.1016/j.tics.2019.12.011.
37. Writing Committee for the ENRICHD Investigators. Effects of Treating Depression and Low Perceived Social Support on Clinical Events after Myocardial Infarction: The Enhancing Recovery in Coronary Heart Disease Patients (ENRICHD) Randomized Trial. *JAMA* ***2003**, 289, 3106–3116*. https://doi.org/10.1001/jama.289.23.3106.
38. Barefoot, J.C.; Burg, M.M.; Carney, R.M.; Cornell, C.E.; Czajkowski, S.M.; Freedland, K.E.; Hosking, J.D.; Khatri, P.; Pitula, C.R.; Sheps, D. Aspects of Social Support Associated With Depression at Hospitalization and Follow-up Assessment Among Cardiac Patients. *J. Cardiopulm. Rehabil.* **2003**, *23*, 404–412. https://doi.org/10.1097/00008483-200311000-00002.
39. Vaglio, J.; Conard, M.; Poston, W.S.; O'Keefe, J.; Haddock, C.K.; House, J.; Spertus, J.A. Testing the performance of the ENRICHD Social Support Instrument in cardiac patients. *Health Qual. Life Outcomes* **2004**, *2*, 24. https://doi.org/10.1186/1477-7525-2-24.
40. Schreurs, P.J.G.; van de Willige, G.; Brosschot, J.F.; Tellegen, B.; Graus, G.M.H. *De Utrechtse Coping Lijst: UCL. Omgaan Met Problemen en Gebeurtenissen*; Swets en Zeitlinger: Lisse, The Netherlands, 1993.
41. Westbrook, M.T.A classification of coping behavior based on multidimensional scaling of similarity ratings. *J. Clin. Psychol.* **1979**, *35*, 407–410. https://doi.org/10.1002/1097-4679(197904)35:2<407:AID-JCLP2270350236>3.0.CO;2-P.
42. Skevington, S.; Lotfy, M.; O'Connell, K. The World Health Organization's WHOQOL-BREF quality of life assessment: Psychometric properties and results of the international field trial. A Report from the WHOQOL Group. *Qual. Life Res.* **2004**, *13*, 299–310. https://doi.org/10.1023/b:qure.0000018486.91360.00.
43. The Whoqol Group. Development of the World Health Organization WHOQOL-BREF Quality of Life Assessment. *Psychol. Med.* **1998**, *28*, 551–558. https://doi.org/10.1017/s0033291798006667.
44. Masthoff, E.D.; Trompenaars, F.J.; Van Heck, G.L.; Hodiamont, P.P.; De Vries, J. Quality of life and psychopathology: Investigations into their relationship. *Aust. N. Z. J. Psychiatry* **2006**, *40*, 333–340. https://doi.org/10.1080/j.1440-1614.2006.01799.x.
